# Virtual 3D models, augmented reality systems and virtual laparoscopic simulations in complicated pancreatic surgeries: state of art, future perspectives, and challenges

**DOI:** 10.1097/JS9.0000000000002231

**Published:** 2025-01-24

**Authors:** Imán Laga Boul-Atarass, Carmen Cepeda Franco, José Domingo Sanmartín Sierra, Javier Castell Monsalve, Javier Padillo Ruiz

**Affiliations:** a Department of Surgery, Virgen del Rocio University Hospital, Seville, Spain; b Oncology Surgery, Cell Therapy, and Organ Transplantation Group, Instituto de Biomedicina de Sevilla (IBiS), University of Sevilla, Seville, Spain; c Electromedical Department, Virgen del Rocio University Hospital, Seville, Spain; d Department of Radiology, Virgen del Rocio University Hospital, Seville, Spain

**Keywords:** 3D printing, augmented reality, digital 3D model, digital twin, pancreatic surgery

## Abstract

Pancreatic surgery is considered one of the most challenging interventions by many surgeons, mainly due to retroperitoneal location and proximity to key and delicate vascular structures. These factors make pancreatic resection a demanding procedure, with successful rates far from optimal and frequent postoperative complications. Surgical planning is essential to improve patient outcomes, and in this regard, many technological advances made in the last few years have proven to be extremely useful in medical fields. This review aims to outline the potential and limitations of 3D digital and 3D printed models in pancreatic surgical planning, as well as the impact and challenges of novel technologies such as augmented/virtual reality systems or artificial intelligence to improve medical training and surgical outcomes.

## Introduction

**Highlights**

- Digital 3D models are a useful tool for preoperative planning in pancreatic surgery.
- 3D printing of digital models provides additional physical cues to understand patients’ anatomy better.
- Augmented reality systems may improve pancreatic surgery outcomes by optimizing the identification of resection lines and healthy anatomical structures.
- Virtual laparoscopic simulations reduce surgeons’ learning curve and lead to better proficiency levels after surgical training.
- Artificial intelligence has wide potential applications pancreatic surgery pre-, intra-, and postoperatively that can positively impact patients’ healthcare.

Over the last few years, powerful resources that traditionally have been applied in engineering fields are slowly finding their way into the grounds of medicine, especially in surgical disciplines. Many surgeons worldwide are starting to benefit during their daily work from 3D models of complex anatomies and augmented reality approaches for preoperative planning in challenging interventions.

Pancreatic cancer (PC) surgery ranks among these interventions as one of the most technically demanding and arduous procedures, requiring in many cases the removal and reconstruction of several digestive organs ^[1,2]^. Since surgical techniques have benefited from significant improvements in the last decades and the mortality rate has dropped to less than 5%^[3]^, postoperative complications have risen as the main threat to patient survival after resection of PC^[4]^.

To reduce them, many factors must be preoperatively considered in an accurate and personalized manner. Two examples would be the tumor location and the degree of vascular compromise since intraoperative blood loss is a common event due to the immediacy of blood vessels^[5]^.

Since many patients are not eligible for surgery because of metastatic spread of the disease or extensive arterial compromise^[6-8]^, the creation of virtual 3D reconstructed models of the tumor and nearby structures allows robust preoperative planning for more aggressive interventions to tackle the most complex cases. The use of 3D models for PC surgery effectively translates into a shortage of surgical time and a reduction of postoperative complications, i.e. pancreatic fistula^[9,10]^.

Current imaging techniques used to determine tumor resectability, such as CT or RMN, usually fall short when it comes to providing an unbiased and thorough knowledge of the anatomical structures since each surgeon performs an internal reconstruction of the 2D images and makes a judgment according to that^[11]^. Virtual 3D models can overcome this subjectivity, especially regarding the arterial vessels, which are the most prone to error area. Moreover, medical students and novice surgeons can use it to perform appropriate risk estimation and planning, with an increased grasp of the anatomical variations and a greater self-confidence.

3D models can also bypass the digital dimension to the physical world through 3D printing, providing surgeons with a valuable opportunity to gain a more tangible knowledge about the spacial relationship between the structures, and even execute surgical maneuvers to mimic the procedure about to be performed in the operating room^[12]^.

In addition to this, focusing on the most recent innovations, augmented reality (AR) and virtual reality (VR) may be some of the biggest technological breakthroughs that surgical medicine has benefited from. On one hand, AR allows the overlapping of a 3D model over the surgical field during the intervention, enabling an easier localization of hidden structures and a reduction of their risk of injury. This image-guided surgery constitutes a helpful resource during complex surgeries for key moments such as the determination of the anatomical position of resection borders^[13,14]^.

On the other hand, VR provides a highly realistic virtual environment in which the surgical team may have a more immersive preoperative planning, and safely improve their skills prior to the real-world case^[15]^. VR for training in surgical settings grants the user the opportunity to walk through the steps of any kind of procedure as much as needed, with integrated instructions and personalized feedback^[16]^ .

In addition to this, laparoscopic surgery is widely used in abdominal surgery, not only because of its inherent reduction in surgical stress but also because of the magnification of the surgical view, although limiting in terms of the surgical field width, it allows the performance of delicate tasks, such as an accurate dissection of lymph nodes in cancer patients^[17,18]^. However, these interventions are mainly performed by experienced surgeons, and having a system that allows proper training as well as an accurate estimation of the surgical skills of young doctors has become a pressing need. Several studies focused on surgeons’ learning curve for laparoscopic duodenopancreatectomy showed that between 60 and 84 cases are required to stabilize their surgical skills and ensure safety^[19,20]^. This long learning curve exposes the imperative need for novel training methodologies. Hand in hand with AR/VR come laparoscopic simulation systems, that mainly aim to tackle the drawback of having a reduced field of view and depth perception during laparoscopic procedures by helping the users to train before surgery and enhance their spacial orientation, while reducing the learning curve of the procedure and increase overall performance^[21]^.

In these virtual environments, artificial intelligence (AI) algorithms have huge relevance, since they excel at tasks related to pattern recognition and can be used for the mass assessment of surgical trainees in virtual reality scenarios^[22,23]^. Moreover, the expansion of AI and its integration with big data analysis systems has encouraged the further development of the so-called “digital twins”^[24]^.

Digital twins in their roots are also virtual 3D models of a real element, but with the additional property of being able to mirror the changes happening in the reality that they represent. The dynamic nature of digital twins is considered the main difference from static virtual 3D models, thanks to a bidirectional flow of information that exists between both the physical and the virtual elements in the digital twin^[24]^. In medicine, the biggest current potential of digital twins can be found in precision medicine and public health. In precision medicine, digital twins can be used to simulate and improve patients’ diagnosis and prognosis, while in public health fields, they can be used for aspects such as tracking personal interactions and the control of epidemics^[25]^. However, their applications in surgical settings are still limited, and further research is still needed^[26]^.

Taking all this into consideration, this review aims to globally synthesize the current applications and prospects of digital and printed models in surgical planning, as well as the impact of groundbreaking developments such as AR or AI in pancreatic surgery, since the complexity of this procedure may benefit greatly from these arising technologies, leading to improved outcomes. Additionally, this review proposes to summarize the main current challenges and limitations of these groundbreaking technologies for a better understanding of the areas where further research is still necessary.

## Preoperative digital and printed 3D models for pancreatic surgery

The medical usefulness of 3D models has been thoroughly explored in the last few years. In a study performed with 15 surgical residents, the researchers compared their preoperative understanding when sketches from CT images were used versus when simulated 3D images were applied. They found out that their preoperative comprehension differed by 30-60%, especially regarding the arterial system, which was presented as the most susceptible to misjudgments^[27]^.

This is one of the many examples that bring to light the huge applicability of 3D models in educational frameworks such as resident programs. Virtual models are a potent tool for information transfer among specialists and residents and an interesting resource for the reduction of the learning curve in challenging surgeries.

**Figure 1.**
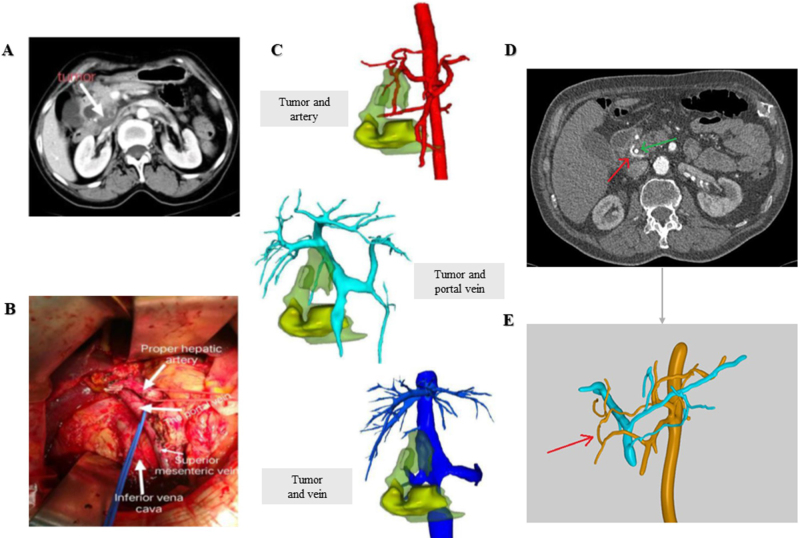
(A) CT scan of the patient); (B) intraoperative image after tumoral resection, with intact SMA, PV, superior mesenteric artery, and IVC; (C) Tumoral relationship with arterial vessels, veins, and portal vein. Images A, B, and C extracted from^[11]^; (D) CT scan showing Bühler anastomosis. Red arrow: vascular variant; green arrow: stent in the hepatic duct; (E) digital 3D model showing the Bühler anastomosis. Images D and E extracted from^[28]^.

Leaving aside the educational purposes of 3D models, studies have shown that their impact on surgeons’ daily practice is not dismissible. In a study with 47 patients undergoing pancreaticoduodenectomy, the cases evaluated with 3D reconstructions rendered a different surgical plan than the one proposed after CT/MRI evaluation. This discrepancy was a consequence of the 3D models successfully exposing the vascular compromise of the patient, therefore allowing an adjustment of the surgical plan (Fig. 1A-C) ^[11]^.

Additionally, another study showed that when the resectability evaluation is executed using virtual 3D reconstructions, parameters such as positive/negative predictive values, sensitivity, or accuracy rendered a value of 100%^[29]^. Moreover, these virtual 3D models have also proven to be a useful tool for the establishment of the tumoral resection line, improving the odds of achieving an effective R0 during surgery^[30]^.

Besides resectability, additional relevant information can be obtained from the virtual models. For instance, tumor volume measurements both preoperatively on a digital model and postoperatively *in vivo* have proven to be statistically equal (t = 0.54, p = 0.59)^[11]^.

Furthermore, another aspect of great importance during PC surgery is the presence of anatomical variants affecting the vascular tree, especially in the hepatobiliary and pancreatic region, that can compromise the outcome of the surgical intervention^[9,31]^.

In this line of thought, some authors aimed to pinpoint blood vessel bifurcation and to identify hepatic artery variations and their possible infiltrations using digital models^[9]^. The presence of these vessel variations, such as uncommon anastomosis, constitutes relevant information to be taken into consideration by surgeons when planning their strategy. A study describes an illustrative example of this situation, in which the CT-angiography showed a patient with a Riolan anastomosis (Fig. 1D and E), while the digital 3D model allowed the preoperative identification of a rare Bühler anastomosis (in only 1–2% of patients), that was intraoperatively confirmed^[28]^.

**Figure 2.**
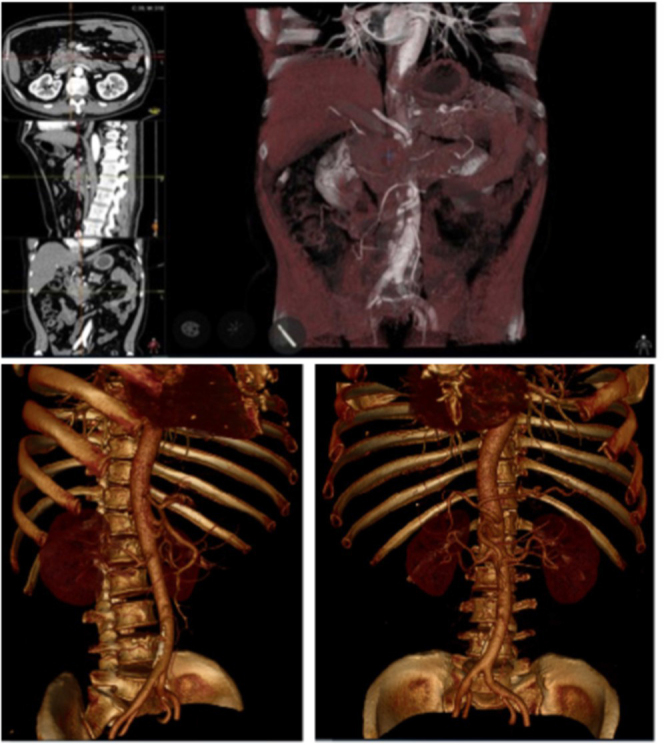
Cinematic rendering of peripancreatic organs and arterial vessels. Image obtained from^[34]^.

In addition to this type of digital model, more realistic virtual representations are obtained with cinematic rendering (Fig. 2), a novel technology capable of providing photorealistic volumes from MR/CT images, with a greater sense of depth and texturing than a standard digital model would^[32,33]^. A study was carried out with surgical residents who were stratified according to their years of training and gender. From them, one group would study a sample case by using only 2D CT images, while the other group would review 2D CT images and a 3D rendered model. After a test evaluating anatomy, diagnosis, tumor stage, and surgical plan, the mean scores obtained were significantly higher in the second group^[34]^.

Although digital 3D models provide significantly more information than 2D medical images, they are limited by their visualization through 2D computer screens. 3D printing constitutes a powerful technology that allows 3D models to leave the digital plane and jump into the physical world, where they provide the user with physical cues and additional dimensional information. Several studies have been performed to assess the suitability of physical 3D models to clinical practice, especially in orthopedic or maxillofacial fields^[35,36]^.

Focusing on abdominal surgery, and more specifically on PC, the available literature is limited, and the utilization of this methodology, although promising, is not as prominent. It has been shown that the tactile involvement resulting from being able to hold a physical 3D model provides a more extensive grasping of the spatial relationships before a splenopancreatectomy intervention^[37,38]^.

Another example is a study where authors aimed to compare digital vs physical models. They showed that the quality of the surgical plan was considerably higher when surgeons reviewed 2D CT images as well as a 3D printed model of a PC patient, compared to those who evaluated 2D CT images and a digital 3D model (76.4 ± 10.5 vs. 66.5 ± 11.2, p = 0.018)^[39]^.

**Figure 3.**
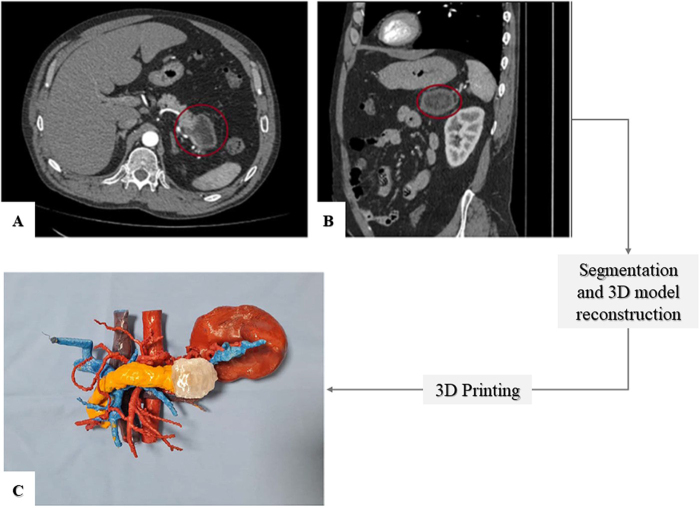
(A) Axial CT scan and (B) sagittal CT scan of a pancreatic cancer patient. Organs; (C) 3D printed model of patient’s anatomy. Image modified from^[38]^.

Additionally, preoperative planning with printed 3D models (Fig. 3) helps anticipate complications and formulate solutions in delicate pathologies. A study reported that surgeons find extremely useful the inclusion of printed 3D models of transparent resin in their preoperative planning so that they can plan the steps to preserve the delicate vascular tree that surrounds the pancreas^[37,38]^.

In cancer surgery, the potential of 3D printed models to ensure proper resection margins by easily determining the boundaries of the tumoral mass relies on the fact that 27% of tumors smaller than 2 cm appear as issoattenuating structures in a CT, undistinguishable from the adjacent pancreatic tissue^[40,41]^. Moreover, since no imaging modality can provide all the information regarding cancer diagnosis, staging, vascular invasion, pancreaticobiliary system evaluation, and presence of metastasis^[42]^, several works have been published proposing a 3D printed model created by the integration of several imaging modalities^[43,44]^. This additional advantage of 3D models would boost the anatomic understanding of the physician.

Besides the many benefits of this technology, there are also important limitations to take into consideration. The most remarkable would be the current 3D printing materials available. Recent advances have been made towards elastomers and silicone^[45,46]^, whose mechanical properties are closer to soft human tissue than the polymers (i.e. polylactic acid or polycaprolactone)^[47]^ that have been traditionally used for medical 3D-printed devices. These materials evoke in surgeons’ tactile sensations similar to real tissue and enable some preoperative rehearsal on aspects such as cutting, suturing, or tumor manipulation, in pursuit of increased R0 resection rates^[37]^.

Additionally, since a digital 3D model is required before the actual printing, image quality is also a factor that influences the final physical model. Moreover, there are also financial costs associated with the printing process, such as the acquisition and maintenance of printing devices and materials that not many healthcare institutions may want to commit to. All of this added to the additional expenses related to the acquirement of the technical skills to use the required software to obtain the final model^[48,49]^, makes the standardization and integration of 3D printing technology in the clinical daily practice a slow process.

The main limitation that this technology presents is the quality of the source images. Poor contrast between the anatomical structures observed in the CT or MRI leads to a model with low resolution and less informational value that will require more manual refinements to obtain a virtual model loyal to the patient’s anatomy^[31]^.

## Digital twins in pancreatic surgery

In the last few years, the concept of digital twins has been applied to many healthcare areas. They were initially developed in the aerospace and aviation industry to test, optimize, and monitor in real-time a physical object or process in the digital world. A digital twin is composed of (1) a physical element in the real world; (2) its digital 3D model; and (3) a bidirectional flow of information or data between them. This last element allows digital twins to dynamically change according to the data input they receive from the reality that they mimic and also allows them to perform real-time simulations and predictions^[50]^. This dynamic trait is the core difference between a digital twin and a static virtual 3D model.

Thanks to the current availability of health data through devices such as activity trackers and sensors, the potential of digital twins in healthcare is also being explored, especially for precision medicine^[51]^. The creation of dynamic models with a wide range of patient data supported with artificial intelligence models can lead to relevant applications, such as in silico clinical trial designs that allow the medical team to test their proposed treatments in a safe and virtual environment^[51,52]^. This can be achieved through the dynamic modeling of complex elements, such as biochemical pathways, tissues, or diseases^[53,54]^.

In surgery, the application of a wide range of technologies to enhance surgeons’ skills and executive abilities (e.g. image-guided surgery, robotic surgery) is generally referred to as computer-assisted surgery (CAS)^[55]^. For some authors, digital twins can be applied to further improve CAS in medical facilities. Their proposed method^[56]^ to achieve this is by combining patient information (physical, physiological, etc.) used to design the digital twin with intraoperative data obtained from several types of sensors (optical trackers, depth sensors, etc.) of both the patient and the surgical scene to update the digital twin in real-time^[57]^.

However, this iterative behavior to constantly improve the digital twin requires high computational costs, and the simulation of the complete physiology of the different tissues is still a challenge to be tackled^[56]^.

The potential use of digital twins has been explored for some surgical interventions to improve outcomes, like in liver^[58]^ or orthopedic surgery^[59]^, as well as some cardiovascular interventions^[60]^. In addition to this, our group is currently developing a digital twin intended to be applied in pancreatic surgery (Project reference EXC-2023-05)

Nevertheless, the application of digital twins in surgery is still limited by the current need for real-time organ modeling that accurately represents real tissue deformation^[26]^. The next sections will discuss the potential applicability of digital twins for preoperative simulations and intraoperative assistance.

## Preoperative virtual simulators and immersive virtual reality (VR) systems for training in laparoscopic pancreatic surgery

Several types of training models can be found (i.e. animal, cadaveric, synthetic or 3D-printed) for a preoperative rehearsal of surgical techniques. From these, the most promising ones would be silicone models, but they do not fully mimic the mechanical properties of the human pancreas (i.e. hardness, fibrosis, elasticity, etc.), and are not completely successful in training in reducing complications, such as pancreatic fistulas^[61,62]^.

However, laparoscopic training based on virtual simulators is the methodology that has proven to lead to greater levels of proficiency than traditional training^[62]^. Since the lack of haptic feedback is one of the biggest drawbacks in laparoscopic surgery, virtual simulators can help surgeons develop visual cues that substitute the tactile stimuli they would normally perceive in open surgery, as well as develop endurance for this physically demanding procedure^[63]^.

In pancreatic surgery, the Whipple procedure is considered by many the most arduous procedure for both patients and the surgical team. Aiming to enhance surgeons’ skills in a risk-free approach, some authors developed a virtual abdominal environment where surgeons can rehearse the intervention by successfully executing the subtasks provided by the simulator^[64]^. These models are developed by creating a mesh with a realistic texturing of the organs of interest (i.e., pancreas and surrounding structures) and with appropriate Young’s modulus and Poisson coefficient to recreate the real mechanical properties of the anatomy^[65]^.

On the other hand, VR systems, first developed in gaming fields, have made the leap to medicine where they take this approach a step further by creating a fully virtual and immersive environment. These scenarios simulate both patients’ anatomy and surgical setting and instrumentation and boost the training and preoperative preparation for better outcomes^[31,66,67]^.

The recent term of surgery digitalization refers to the process of creating a digital twin that accurately replicates the physical entities involved in a surgical intervention, as well as their interactions (devices, medical specialists, patient, etc.) using scanners, sensors and medical imaging techniques (endoscopy, MIR, CT, ultrasound, etc.) as data sources. This can be very useful for preoperative rehearsal in a hyper-realistic environment, and for the assessment of surgical performance^[68]^, and some authors are exploring its potential use for simulating minimally invasive robotic surgeries^[69]^.

However, although highly appreciated by experienced surgeons^[31]^, this groundbreaking technology is not fully integrated yet because it requires trained medical staff and investment in hospital resources and more time is needed for a better consolidation of these technologies.

## Intraoperative assisted pancreatic surgery with augmented reality systems

**Figure 4.**
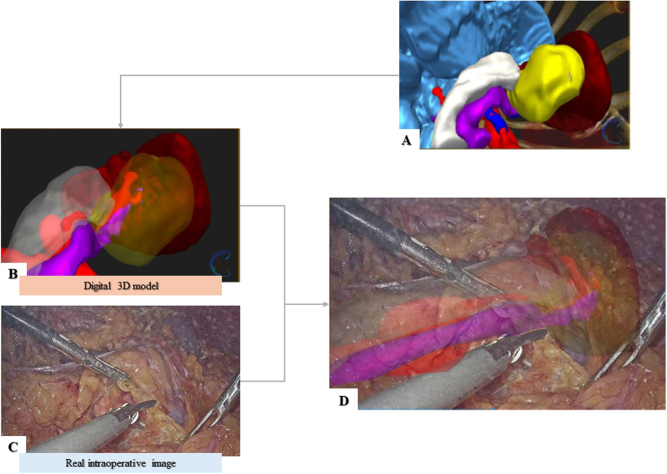
(A) and (B) Digital 3D model of patient’s anatomy with different levels of transparency; (C) Intraoperative image of the surgical field; (D) Overlapping of digital model and real image. Image modified from^[21]^.

Once a digital 3D model is available, its applicability is far from limited to 3D printing. In the last few years, operation rooms have witnessed the introduction of novel AR navigation systems in the surgical setting^[70]^. This technology is based on the superimposition of a semitransparent digital model on a real image in the surgical field and provides surgeons with guidance during their interventions to locate concealed anatomical structures^[71,72]^. AR has proved to be helpful in pancreaticoduodenectomy for the identification of the dissection lines around the superior mesenteric artery while preserving surrounding healthy structures (Fig. 4) ^[73]^.

**Figure 5.**
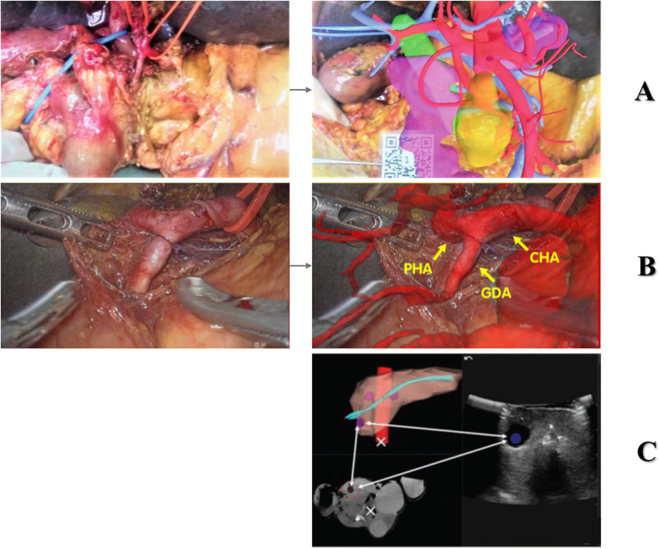
(A) Intraoperative view of surgical field and model registration using a QR code (image obtained from^[72]^); (B) intraoperative view of surgical field and model registration using anatomical markers (image obtained from^[76]^); (C) Landmark identification in a CT scan, ultrasound and 3D model (image obtained from^[77]^).

Registration refers to the critical process of achieving accurate anatomic correspondence and alignment between the virtual structures of the 3D model and the real image of the patient. To date, this process is not yet extensively automatized^[71]^, and it is performed manually (i.e. using QR code scanning (Fig. 5A)^[72]^, but the natural deformation and movement of the patient’s anatomy cause some displacement between the virtual and the real images. A procedure to partially overcome this sensitivity to virtual vs real incongruence is to take advantage of anatomical landmarks (Fig. 5B). The identification of the anatomical markers in both the medical images (i.e. CT scan or ultrasound; Fig. 5C) and in the digital 3D model allows the fuse of the preoperative images in the real world^[74]^. This approach achieves a registration error lower than 5 mm, as proved in a study where AR assisted in the ligation of the inferior pancreaticoduodenal artery and the identification of lesions and resection lines in 24 patients^[75]^ after a successful fiducial registration process. Moreover, AR surgical navigation systems can be also used for the training of young surgeons and in the standardization of surgical procedures^[71,73]^.

Despite this technical limitation related to the registration process, some authors report that pancreatic surgery, as difficult as it is, may benefit from AR surgical navigation systems with anatomical fiducial markers more than other abdominal interventions, due to the retroperitoneal location of the pancreas, which constrains the organ and blocks its deformity^[78]^.

Following the Miami guidelines, pancreatic surgery is being extensively performed by laparoscopic approaches that seem to render lower complication rates than open surgeries^[79]^. A recent study reported a novel automatic image registration for laparoscopic interventions, but further tests are required to evaluate its accuracy^[21]^. In addition to this, there are some challenges associated, such as visual field obstruction, lack of haptic feedback, and a higher risk of confusing important landmarks in the patient’s anatomy. Some authors propose the use of virtual 3D endoscopic imaging as guidance during surgery using the laparoscopic point of view and allowing the manipulation of the 3D image in a different monitor to recognize and avoid difficult areas^[80]^.

In addition to this, the integration of digital twins with intraoperative augmented reality systems has been explored by some authors in other surgical fields, who tried to merge a digital twin with a holographic AR navigation system^[58]^. Another ongoing project is aiming to develop a prototype of digital twin combining AR systems and artificial intelligence to show the potential capabilities of these systems in intraoperative surgical settings^[81]^.

Although the potential of AR in surgery is far from limited, the obstacles that restrain this technology don’t fall behind. One of the biggest challenges is to achieve organ deformity as the surgery evolves^[82]^. Additionally, some authors are focusing on achieving automatic registration systems that accommodate the natural deformations that occur during surgery^[72,82]^. The lack of real-time performance and accuracy, as well as the difficulty regarding the software management of organ distortions and their ability to relocate displaced elements remain the biggest barriers to digital twins implementation in surgery^[58,81]^ .

## Postoperative utility of digital 3D models and VR in pancreatic surgery

The use of digital 3D models and VR technologies in postoperative follow-ups after surgery is an emerging field that could play an important role in preventing and potentially managing complications. Similarly to what we previously described for the preoperative approach, digital 3D models could be useful for differentiating between tumor recurrence or postoperative inflammatory reaction, helping the decision in these complicated patients.

In addition to this potential application, some specific postoperative utilities have been proposed, such as the accurate evaluation of postoperative anatomy, and the identification of complications (i.e., pancreatic fistula or abscess). Along these lines, some authors^[83]^ demonstrated that three-dimensional simulation of pancreatic parenchyma, pancreatic ducts, and vascular arrangement using deep learning algorithms is useful for postoperative visualization and evaluation. Additionally, other authors have explored the postoperative use of 3D models in renal cancer patients, although their work could be also applied to other types of malignancies, such as PC. These authors found that digital 3D models significantly improved postoperative communication with patients, and helped with an early detection of some complications^[84]^.

On the other hand, VR could also have some applications in postoperative follow-up. VR can facilitate the creation of an interactive platform for the review of postoperative images in an immersive and multi-operational environment, which would enable the early detection of complications, and thus a quick intervention plan, if necessary. Although most current applications of VR focus on preoperative planning^[85]^, the same technology can be adapted for postoperative monitoring, potentially improving results.

To sum up, further studies are required with the current developments of both 3D models and VR to improve the precision when detecting possible complications after pancreas surgery and, at the same time, to facilitate surgical planning before a potential intervention in these patients.

## Artificial intelligence applications in pancreatic surgery

Artificial intelligence (AI), originated in the first half of the 20th century^[86]^ on computer science fields, has spread like wildfire to many areas of human life in the last decades. AI is considered a term to refer to algorithms that emulate human thinking while taking advantage of computational technologies that allow fast and accurate performance of repetitive tasks. Many countries are trying to promote its development through programs like the European AI-BOOST challenge^[87]^, which aims to further develop AI and safely adopt it in our society.

AI integration in surgery encompasses vast tasks, such as diagnosis and risk predictions, management of resources, or patient selection^[88,89]^. Focusing on preoperative planning in pancreatic surgery, AI can be applied pre-, intra-, and postoperatively at several levels.

Preoperatively, surgical teams can make AI-guided resectability decisions from CT images^[77]^. A previous study was published proposing a model that reported 100% accuracy in its treatment predictions^[29]^. Although it is far from perfect, since some vascular compressions were misinterpreted as tumoral invasions by the model, it is a promising resource that can positively impact the surgical opportunities of PC patients. The AI models have the potential to allow accurate discrimination between benign and malignant lesions, which can make the difference between surgery and surveillance^[77]^.

Regarding AI in preoperative 3D printing, the main use would be workflow automatization, firstly by achieving an automatic segmentation of a patient’s anatomy, and afterward by optimizing the printing process through the selection of the best parameters to prevent faulty physical models^[90]^.

Intraoperatively, AI applications are also remarkable. Since the spread of minimally invasive surgery, the term “surgomics”^[91]^ has emerged as a reference to all the data that can be collected during surgery, such as surgical field information obtained from the laparoscopic/endoscopic camera or patient’s physiological values. This information can be processed by AI systems for surgical training, performance evaluation, or decision support to the surgical team. Moreover, real-time registration between virtual 3D models and patient’s anatomy can be greatly optimized with AI approaches^[77]^.

Postoperative applications of AI can be divided into complications predictions right after the intervention and prognostic estimations. Thanks to the increasing amount of data, AI algorithms can be trained to predict complications such as postoperative pancreatic fistula or bleeding^[92-94]^. On the other hand, personalized surveillance and prognostic estimations of intervened patients can be more exhaustive and accurate with AI models, although to date no have been found in the available literature regarding the surveillance use of AI after PC resection^[77]^, but there are for other pathologies (i.e. prostate cancer or lung cancer)^[95,96]^.

A form of AI is the so-called “generative AI” (GAI), which can generate a wide variety of content such as text, images, or animations, in response to human users’ prompts after being trained on large amounts of data^[97]^. A few examples of GAI are ChatGPT, Gemini, or DALL-E. For healthcare applications, current trends are moving towards the training of these GAIs with patients’ clinical data^[97]^.

In surgical settings, the applicability of GAIs can be observed at a pre-, intra-, and postoperative level. Both pre- and postoperatively, a cross-sectional study showed that GAIs in the form of chatbots can render empathetic responses to patients’ questions^[98]^. These results open a window of possibility towards the potential use of GAI for generating drafts of medical responses and alleviating medical staff workload, also by providing patients with personalized recovery guidance and medication schedules^[99]^.

Moreover, some preoperative application in surgery could be its use as a prehospital assessment tool to evaluate the symptoms and flag high-risk patients^[100]^. Additional potential applicability is its integration into clinical decision processes during the surgical planning step through the interpretation of patients’ data and the generation of clinical reports or even image simulations of the possible outcome of the intervention^[101]^. Is proposed by some authors that its ability to process huge amounts of medical data may be useful to generate artificial treatment plants, patient histories, or even virtual 3D models for surgical planning^[102]^.

GAI-based simulations are also being considered for preoperative surgical training, taking advantage of the hyperrealism of the environment that these technologies generate^[99]^.

Intraoperatively, GAIs could have vast applications, such as the generation of intraoperative reports during the intervention from dictation of the medical stuff, therefore reducing the time required for this task traditionally performed manually, or even the integration of patients’ images from diverse inputs in the surgical field^[101]^.

Among the concerns of GAI usage in healthcare, there is the possible “hallucination” effect, in which the GAI fabricates answers when it does not dispose of enough information^[97]^ .This raises ethical questions regarding the accuracy and authenticity of AI-generated medical information, and current research is focusing on the development of checkpoints where the verity of the information can be assessed during the generation process by the GAI^[102]^, as well as in the establishment of boundaries to prevent AI from compromising specialists’ judgment and patients’ safety^[99]^.

In light of all of the above, it is evident the great potential of AI in healthcare applications, although further developments are required to make them more user-friendly and achieve international agreements regarding patients’ data management and safety.

## Virtual models, digital twins, simulators, augmented reality systems and AI: specific proposals in surgery

Virtual 3D models have a huge potential for preoperative planning in pancreatic surgery, more specifically in tasks related to medical training, tumor resectability evaluation, possible resection line formulation, and tumoral measurements, as well as the evaluation of blood vessel compromise and anatomical variations.

In addition to this, 3D printing is proposed to assist during surgical planning by providing physical cues and a clearer spatial relationship between the anatomical structures, as well as by potentially improving the determination of the resection margins and the preservation of vascular structures.

Considering this, VR is proposed as a powerful resource to integrate with laparoscopic simulations and digital twins, which may optimize the learning process of surgeons and residents, and provide a realistic and risk-free environment for surgical rehearsal.

On the other hand, AR systems in pancreatic surgery seem to be able to fulfill several purposes, such as the provision of visual representation of resection lines in the surgical field or the identification of hidden anatomical structures. When combined with digital twins, they have the potential to optimize surgical interventions and patient outcomes.

Finally, AI has great potential in healthcare applications for relevant tasks such as AI-guided clinical decisions, malignancy discrimination, process automatization, prognostic estimations, or personalized surveillance.

## Conclusions

Virtual and printed 3D models are nowadays being integrated into surgical environments at a steady pace, especially for preoperative planning in complex interventions such as pancreatic surgery. In addition to this, the newest technologies, such as digital twins, AR, VR, and AI, although still not very notable, constitute a promising resource that may positively impact surgical performance in PC surgeries in which both preoperative and intraoperative application of these technologies could have a positive impact in oncologic surgical outcome.

## Data Availability

Data sharing is not applicable to this article.

## Research registration unique identifying number (UIN)

Not applicable. No human subjects were involved in this study.

## References

[1] Bishop MA Simo K. Pancreatectomy. 2024. 33231979

[2] Giuliano K Ejaz A He J. Technical aspects of pancreaticoduodenectomy and their outcomes. Chin Clin Oncol 2017;6:64–64. 29156887 10.21037/cco.2017.09.01

[3] Wegner RE Verma V Hasan S. Incidence and risk factors for post-operative mortality, hospitalization, and readmission rates following pancreatic cancer resection. J Gastrointest Oncol 2019;10:1080–93. 31949925 10.21037/jgo.2019.09.01 PMC6955019

[4] Mintziras I Wächter S Manoharan J Kanngiesser V Maurer E Bartsch DK. Postoperative morbidity following pancreatic cancer surgery is significantly associated with worse overall patient survival; systematic review and meta-analysis. Surg Oncol 2021;38:101573. 33857838 10.1016/j.suronc.2021.101573

[5] Tamagawa H Aoyama T Yamamoto N. The impact of intraoperative blood loss on the survival of patients with stage II/III pancreatic cancer. Vivo (Brooklyn) 2020;34:1469–74. 10.21873/invivo.11931 PMC7279845 32354948

[6] Evans DB. What makes a pancreatic cancer resectable? Am Soc Clin Oncol Educat Book 2018;38:300–05. 10.1200/EDBK_200861 30231408

[7] Reames BN Blair AB Krell RW. Management of locally advanced pancreatic cancer. Ann Surg 2021;273:1173–81. 31449138 10.1097/SLA.0000000000003568

[8] Miao Y Cai B Lu Z. Technical options in surgery for artery-involving pancreatic cancer: invasion depth matters. Surg Open Sci 2023;12:55–61. 36936450 10.1016/j.sopen.2023.03.001 PMC10020102

[9] Yang J Fang C-H Fan Y-F. To assess the benefits of medical image three-dimensional visualization system assisted pancreaticoduodenctomy for patients with hepatic artery variance. Int J Med Robot 2014;10:410–17. 24711375 10.1002/rcs.1590

[10] Zhang W Cai W He B Xiang N Fang C Jia F. A radiomics-based formula for the preoperative prediction of postoperative pancreatic fistula in patients with pancreaticoduodenectomy. Cancer Manag Res 2018;10:6469–78. 30568506 10.2147/CMAR.S185865 PMC6276820

[11] Zhang Y Yang G Lei P Zhang D. Clinical application of 3D visualization technology in pancreatoduodenectomy. Surg Tech Dev 2022;11:90–97.

[12] Pietrabissa A Marconi S Negrello E. An overview on 3D printing for abdominal surgery. Surg Endosc 2020;34:1–13. 10.1007/s00464-019-07155-5 31605218

[13] Dai J Qi W Qiu Z Li C. The application and prospection of augmented reality in hepato-pancreato-biliary surgery. Biosci Trends 2023;17:2023.01086. 10.5582/bst.2023.01086 37357403

[14] Rad AA Vardanyan R Lopuszko A. Virtual and augmented reality in cardiac surgery. Braz J Cardiovasc Surg 2022;37:123–127. 34236814 10.21470/1678-9741-2020-0511 PMC8973146

[15] Laspro M Groysman L Verzella AN Kimberly LL Flores RL. The use of virtual reality in surgical training: implications for education. Patient Safety, and Global Health Equity Surgeries 2023;4:635–46.

[16] Kuhn AW Yu JK Gerull KM Silverman RM Aleem AW. Virtual reality and surgical simulation training for orthopaedic surgery residents. JBJS Open Access 2024;9:e23. 10.2106/JBJS.OA.23.00142 PMC10950179 38511201

[17] Nomura T Mamada Y Nakamura Y. Laparoscopic skill improvement after virtual reality simulator training in medical students as assessed by augmented reality simulator. Asian J Endosc Surg 2015;8:408–12. 26216064 10.1111/ases.12209

[18] Kanaya S Haruta S Kawamura Y. Laparoscopy distinctive technique for suprapancreatic lymph node dissection: medial approach for laparoscopic gastric cancer surgery. Surg Endosc 2011;25:3928–29. 21660629 10.1007/s00464-011-1792-0

[19] Kim S Yoon Y-S Han H-S Cho JY Choi Y Lee B. Evaluation of a single surgeon’s learning curve of laparoscopic pancreaticoduodenectomy: risk-adjusted cumulative summation analysis. Surg Endosc 2021;35:2870–78. 32548654 10.1007/s00464-020-07724-z

[20] Braga M Ridolfi C Balzano G Castoldi R Pecorelli N Di Carlo V. Learning curve for laparoscopic distal pancreatectomy in a high-volume hospital. Updates Surg 2012;64:179–83. 22763577 10.1007/s13304-012-0163-2

[21] Du C Li J Zhang B Feng W Zhang T Li D. Intraoperative navigation system with a multi-modality fusion of 3D virtual model and laparoscopic real-time images in laparoscopic pancreatic surgery: a preclinical study. BMC Surg 2022;22:139. 35410155 10.1186/s12893-022-01585-0 PMC9004060

[22] Belmar F Gaete MI Escalona G. Artificial intelligence in laparoscopic simulation: a promising future for large-scale automated evaluations. Surg Endosc 2023;37:4942–46. 36192656 10.1007/s00464-022-09576-1 PMC9529161

[23] Vedula SS Ghazi A Collins JW. Artificial intelligence methods and artificial intelligence-enabled metrics for surgical education: a multidisciplinary consensus. J Am Coll Surg 2022;234:1181–92. 35703817 10.1097/XCS.0000000000000190 PMC10634198

[24] Armeni P Polat I De Rossi LM Diaferia L Meregalli S Gatti A. Digital twins in healthcare: is it the beginning of a new era of evidence-based medicine? a critical review. J Pers Med 2022;12:1255. 36013204 10.3390/jpm12081255 PMC9410074

[25] Kamel Boulos MN Zhang P. Digital twins: from personalised medicine to precision public health. J Pers Med 2021;11:745. 34442389 10.3390/jpm11080745 PMC8401029

[26] Ahmed H Devoto L. The potential of a digital twin in surgery. Surg Innov 2021;28:509–10. 33290181 10.1177/1553350620975896 PMC8381595

[27] Miyamoto R Oshiro Y Nakayama K Ohkohchi N. Impact of three-dimensional surgical simulation on pancreatic surgery. Gastrointest Tumors 2017;4:84–89. 29594109 10.1159/000484894 PMC5869364

[28] Templin R Tabriz N Hoffmann M. Case report: virtual and interactive 3D vascular reconstruction before planned pancreatic head resection and complex vascular anatomy: a bench-to-bedside transfer of new visualization techniques in pancreatic surgery. Front Surg 2020;7:38. 32626723 10.3389/fsurg.2020.00038 PMC7314924

[29] Fang C Zhu W Wang H. A new approach for evaluating the resectability of pancreatic and periampullary neoplasms. Pancreatology 2012;12:364–71. 22898639 10.1016/j.pan.2012.05.006

[30] Abe Y Itano O Kitago M. Computer assisted surgery, preoperative planning and navigation for pancreatic cancer. J Hepatobiliary Pancreat Sci 2014;21:251–55. 24520054 10.1002/jhbp.84

[31] Sampogna G Pugliese R Elli M Vanzulli A Forgione A. Routine clinical application of virtual reality in abdominal surgery. Minimally Invasive Ther Allied Technol 2017;26:135–43. 10.1080/13645706.2016.1275016 28084141

[32] Javed AA Young RWC Habib JR. Cinematic rendering: novel tool for improving pancreatic cancer surgical planning. Curr Probl Diagn Radiol 2022;51:878–83. 35595587 10.1067/j.cpradiol.2022.04.001

[33] Stott M Kausar A. Can 3D visualisation and navigation techniques improve pancreatic surgery? A systematic review. Artificial Intelligence Surgery 2023;3:207–16.

[34] Lin C Gao J Zheng H. Three-dimensional visualization technology used in pancreatic surgery: a valuable tool for surgical trainees. J Gastrointestinal Surg 2020;24:866–73. 10.1007/s11605-019-04214-z PMC7165138 31012044

[35] Dadhich A Nilesh K Shah S Saluja H. Three-dimensional printing in maxillofacial surgery: a quantum leap in future. Natl J Maxillofac Surg 2022;13:203. 10.4103/njms.NJMS_65_20 PMC9651252 36393959

[36] Wixted CM Peterson JR Kadakia RJ Adams SB. Three-dimensional printing in orthopaedic surgery: current applications and future developments. J Am Acad Orthop Surg Glob Res Rev 2021;5:e20.00230–11. 33877073 10.5435/JAAOSGlobal-D-20-00230 PMC8059996

[37] Song C Min JH Jeong WK. Use of individualized 3D-printed models of pancreatic cancer to improve surgeons’ anatomic understanding and surgical planning. Eur Radiol 2023;33:7646–55. 37231071 10.1007/s00330-023-09756-0

[38] Arsenkov S Plavevski O Nikolovski A Arsenkov L Shurlani A Saliu V. Enhancing surgical planning of distal splenopancreatectomy through 3D printed models: a case report. J Surg Case Rep 2023;9:rjad528. 10.1093/jscr/rjad528 PMC10506889 37727227

[39] Zheng Y Yu D Zhao J Wu Y Zheng B. 3D printout models vs. 3D-rendered images: which is better for preoperative planning? J Surg Educ 2016;73:518–23. 26861582 10.1016/j.jsurg.2016.01.003

[40] Vernuccio F Messina C Merz V Cannella R Midiri M. Resectable and borderline resectable pancreatic ductal adenocarcinoma: role of the radiologist and oncologist in the era of precision medicine. Diagnostics 2021;11:2166. 34829513 10.3390/diagnostics11112166 PMC8623921

[41] Elbanna KY Jang H-J Kim TK. Imaging diagnosis and staging of pancreatic ductal adenocarcinoma: a comprehensive review. Insights Imaging 2020;11:58. 32335790 10.1186/s13244-020-00861-y PMC7183518

[42] Chen F-M Ni J-M Zhang Z-Y Zhang L Li B Jiang C-J. Presurgical evaluation of pancreatic cancer: a comprehensive imaging comparison of CT versus MRI. Am J Roentgenol 2016;206:526–35. 26901008 10.2214/AJR.15.15236

[43] Pereira da Silva N Abreu I Serôdio M Ferreira L Alexandrino H Donato P. Advanced hepatic vasculobiliary imaging segmentation and 3D reconstruction as an aid in the surgical management of high biliary stenosis. BMC Med Imaging 2020;20:120. 33092546 10.1186/s12880-020-00520-0 PMC7584102

[44] Gosnell J Pietila T Samuel BP Kurup HKN Haw MP Vettukattil JJ. Integration of computed tomography and three-dimensional echocardiography for hybrid three-dimensional printing in congenital heart disease. J Digit Imaging 2016;29:665–69. 27072399 10.1007/s10278-016-9879-8 PMC5114226

[45] Duran MM Moro G Zhang Y Islam A. 3D printing of silicone and polyurethane elastomers for medical device application: a review. Adv Ind Manuf Eng 2023;7:100125.

[46] Hatamikia S Jaksa L Kronreif G. Silicone phantoms fabricated with multi-material extrusion 3D printing technology mimicking imaging properties of soft tissues in CT. Z Med Phys 2023. doi:10.1016/j.zemedi.2023.05.007. PMC12166924 37380561

[47] Appuhamillage GA Ambagaspitiya SS Dassanayake RS Wijenayake A. 3D and 4D printing of biomedical materials: current trends, challenges, and future outlook. Explor Med 2024;5:17–47.

[48] Godinho MR Mestrinho LA. In-house three-dimensional printing for surgical planning: learning curve from a case series of temporomandibular joint and related disorders. Front Vet Sci 2024;11:1347107. 38379923 10.3389/fvets.2024.1347107 PMC10876850

[49] Kim J-Y Lee Y-C Kim S-G Garagiola U. Advancements in oral maxillofacial surgery: a comprehensive review on 3D printing and virtual surgical planning. Appl Sci 2023;13:9907.

[50] Mollica L Leli C Sottotetti F Quaglini S Locati LD Marceglia S. Digital twins: a new paradigm in oncology in the era of big data. ESMO Real World Data Digital Oncol 2024;5:100056. 10.1016/j.esmorw.2024.100056 PMC12836754 41648658

[51] Katsoulakis E Wang Q Wu H. Digital twins for health: a scoping review. NPJ Digit Med 2024;7:77. 38519626 10.1038/s41746-024-01073-0 PMC10960047

[52] Moingeon P Chenel M Rousseau C Voisin E Guedj M. Virtual patients, digital twins and causal disease models: paving the ground for in silico clinical trials. Drug Discov Today 2023;28:103605. 37146963 10.1016/j.drudis.2023.103605

[53] Zhang K Zhou H-Y Baptista-Hon DT. Concepts and applications of digital twins in healthcare and medicine. Patterns 2024;5:101028. 39233690 10.1016/j.patter.2024.101028 PMC11368703

[54] Sun T He X Li Z. Digital twin in healthcare: recent updates and challenges. Digit Health 2023;9. 10.1177/20552076221149651 PMC9830576 36636729

[55] Giménez M Gallix B Costamagna G. Definitions of computer-assisted surgery and intervention, image-guided surgery and intervention, hybrid operating room, and guidance systems. Ann Surg Open 2020;1:e021. 10.1097/AS9.0000000000000021 PMC7771637 33392607

[56] Qin J Wu J. Realizing the potential of computer-assisted surgery by embedding digital twin technology. JMIR Med Inform 2022;10:e35138. 36346669 10.2196/35138 PMC9682458

[57] Ding H Seenivasan L Killeen BD Cho SM Unberath M. Digital twins as a unifying framework for surgical data science: the enabling role of geometric scene understanding. Artif Intel Surg 2024;4:109–38.

[58] Shi Y Deng X Tong Y. Synergistic digital twin and holographic augmented-reality-guided percutaneous puncture of respiratory liver tumor. IEEE Trans Hum Mach Syst 2022;52:1364–74.

[59] Hernigou P Olejnik R Safar A Martinov S Hernigou J Ferre B. Digital twins, artificial intelligence, and machine learning technology to identify a real personalized motion axis of the tibiotalar joint for robotics in total ankle arthroplasty. Int Orthop 2021;45:2209–17. 34351462 10.1007/s00264-021-05175-2

[60] Poletti G Antonini L Mandelli L. Towards a digital twin of coronary stenting: a suitable and validated image-based approach for mimicking patient-specific coronary arteries. Electronics (Basel) 2022;11:502.

[61] Joshi K Espino DM Shepherd DET. Pancreatic anastomosis training models: current status and future directions. Pancreatology 2024;24:624–29. 38580492 10.1016/j.pan.2024.03.020

[62] Yang J Luo P Wang Z Shen J. Simulation training of laparoscopic pancreaticojejunostomy and stepwise training program on a 3D-printed model. Int J Surg 2022;107:106958. 36283653 10.1016/j.ijsu.2022.106958

[63] Haney CM Karadza E Limen EF. Training and learning curves in minimally invasive pancreatic surgery: from simulation to mastery. J Pancreatol 2020;3:101–10.

[64] Demirel D Yu A Halic T Kockara S Web based camera navigation for virtual pancreatic cancer surgery: whipple surgery simulator (VPanSS). 2014 IEEE Innovations in Technology Conference, IEEE; 2014, p. 1–8. doi:10.1109/InnoTek.2014.6877375.

[65] Mena A Bel D Alfaro I González D Cueto E Chinesta F. Towards a pancreatic surgery simulator based on model order reduction. Adv Model Simul Eng Sci 2015;2:31.

[66] Ochs V Saad B Taha-Mehlitz S. An analysis of virtual reality in abdominal surgery – a scoping review. Int J Med Robot 2024;20:e2623. 38375774 10.1002/rcs.2623

[67] Parham G Bing EG Cuevas A. Creating a low-cost virtual reality surgical simulation to increase surgical oncology capacity and capability. Ecancermedicalscience 2019;13:910. 31123493 10.3332/ecancer.2019.910 PMC6445537

[68] Hein J Giraud F Calvet L Creating a digital twin of spinal surgery: a proof of concept. 2024 IEEE/CVF Conference on Computer Vision and Pattern Recognition Workshops (CVPRW), IEEE; 2024, p. 2355–64. doi:10.1109/CVPRW63382.2024.00241.

[69] Cai X Wang Z Li S Pan J Li C Tai Y. Implementation of a virtual reality based digital-twin robotic minimally invasive surgery simulator. Bioengineering 2023;10:1302. 38002426 10.3390/bioengineering10111302 PMC10669730

[70] Seetohul J Shafiee M Sirlantzis K. Augmented reality (AR) for surgical robotic and autonomous systems: state of the art, challenges, and solutions. Sensors 2023;23:6202. 37448050 10.3390/s23136202 PMC10347167

[71] Cremades Pérez M Espin Álvarez F Pardo Aranda F. Augmented reality in hepatobiliary-pancreatic surgery: a technology at your fingertips. Cirugía Española (English Edition) 2023;101:312–18. 10.1016/j.cireng.2023.02.004 36781048

[72] Tang R Yang W Hou Y. Augmented reality-assisted pancreaticoduodenectomy with superior mesenteric vein resection and reconstruction. Gastroenterol Res Pract 2021;2021:1–7. 10.1155/2021/9621323 PMC7990556 33815500

[73] Marzano E Piardi T Soler L. Augmented reality-guided artery-first pancreatico-duodenectomy. J Gastrointestinal Surg 2013;17:1980–83. 10.1007/s11605-013-2307-1 23943389

[74] Müller PC Haslebacher C Steinemann DC. Image-guided minimally invasive endopancreatic surgery using a computer-assisted navigation system. Surg Endosc 2021;35:1610–17. 32253555 10.1007/s00464-020-07540-5

[75] Okamoto T Onda S Yasuda J Yanaga K Suzuki N Hattori A. Navigation surgery using an augmented reality for pancreatectomy. Dig Surg 2015;32:117–23. 25766302 10.1159/000371860

[76] Wu X Wang D Xiang N. Augmented reality-assisted navigation system contributes to better intraoperative and short-time outcomes of laparoscopic pancreaticoduodenectomy: a retrospective cohort study. Int J Surg 2023;109:2598–607. 37338535 10.1097/JS9.0000000000000536 PMC10498855

[77] Kuemmerli C Rössler F Berchtold C. Artificial intelligence in pancreatic surgery: current applications. J Pancreatol 2023;6:74–81.

[78] Onda S Okamoto T Kanehira M. Identification of inferior pancreaticoduodenal artery during pancreaticoduodenectomy using augmented reality-based navigation system. J Hepatobiliary Pancreat Sci 2014;21:281–87. 23970384 10.1002/jhbp.25

[79] Asbun HJ Moekotte AL Vissers FL. The Miami international evidence-based guidelines on minimally invasive pancreas resection. Ann Surg 2020;271:1–14. 31567509 10.1097/SLA.0000000000003590

[80] Aoki T Koizumi T Mansour DA. Virtual reality with three-dimensional image guidance of individual patients’ vessel anatomy in laparoscopic distal pancreatectomy. Langenbecks Arch Surg 2020;405:381–89. 32410077 10.1007/s00423-020-01871-6

[81] Riedel P Riesner M Wendt K Assmann U Data-driven digital twins in surgery utilizing augmented reality and machine learning. 2022 IEEE International Conference on Communications Workshops (ICC Workshops), IEEE; 2022, p. 580–85. doi:10.1109/ICCWorkshops53468.2022.9814537.

[82] Shibuya S Shido N Shirai R. Proposal of simulation-based surgical navigation and development of laparoscopic surgical simulator that reflects motion of surgical instruments in real-world. Int J Autom Tech 2023;17:262–76.

[83] Miyamoto R Takahashi A Ogasawara A. Three-dimensional simulation of the pancreatic parenchyma, pancreatic duct and vascular arrangement in pancreatic surgery using a deep learning algorithm. PLoS One 2022;17:e0276600. 36306322 10.1371/journal.pone.0276600 PMC9616217

[84] Tang G Liu H Wang X. The role of three-dimensional models in preoperative communication and postoperative management of partial nephrectomy. Asia Pac J Oncol Nurs 2023;10:100222. 37181815 10.1016/j.apjon.2023.100222 PMC10173163

[85] Lyuksemburg V Abou-Hanna J Marshall JS. Virtual reality for preoperative planning in complex surgical oncology: a single-center experience. J Surg Res 2023;291:546–56. 37540972 10.1016/j.jss.2023.07.001

[86] Anyoha R. The history of artificial intelligence 2017. accessed July 1, 2024. https://sitn.hms.harvard.edu/flash/2017/history-artificial-intelligence/.

[87] Delivering the next level of European AI open competitions; n.d. accessed July 1, 2024. https://aiboost-project.eu/

[88] Guni A Varma P Zhang J Fehervari M Ashrafian H. Artificial intelligence in surgery: the future is now. Eur Surg Res 2024;65:22–39. 10.1159/000536393 38253041

[89] Varghese C Harrison EM O’Grady G Topol EJ. Artificial intelligence in surgery. Nat Med 2024;30:1257–68. 38740998 10.1038/s41591-024-02970-3

[90] Bari H Wadhwani S Dasari BVM. Role of artificial intelligence in hepatobiliary and pancreatic surgery. World J Gastrointest Surg 2021;13:7–18. 33552391 10.4240/wjgs.v13.i1.7 PMC7830072

[91] Wagner M Brandenburg JM Bodenstedt S. Surgomics: personalized prediction of morbidity, mortality and long-term outcome in surgery using machine learning on multimodal data. Surg Endosc 2022;36:8568–91. 36171451 10.1007/s00464-022-09611-1 PMC9613751

[92] Schlanger D Graur F Popa C Moiș E Al Hajjar N. The role of artificial intelligence in pancreatic surgery: a systematic review. Updates Surg 2022;74:417–29. 35237939 10.1007/s13304-022-01255-z

[93] Han IW Cho K Ryu Y. Risk prediction platform for pancreatic fistula after pancreatoduodenectomy using artificial intelligence. World J Gastroenterol 2020;26:4453–64. 32874057 10.3748/wjg.v26.i30.4453 PMC7438201

[94] Wakiya T Ishido K Kimura N. Prediction of massive bleeding in pancreatic surgery based on preoperative patient characteristics using a decision tree. PLoS One 2021;16:e0259682. 34752505 10.1371/journal.pone.0259682 PMC8577735

[95] Nayan M Salari K Bozzo A. A machine learning approach to predict progression on active surveillance for prostate cancer. J Urol Oncol 2022;40:161.e1–161.e7. 10.1016/j.urolonc.2021.08.007 PMC8882704 34465541

[96] Hindocha S Charlton TG Linton-Reid K. A comparison of machine learning methods for predicting recurrence and death after curative-intent radiotherapy for non-small cell lung cancer: development and validation of multivariable clinical prediction models. EBioMedicine 2022;77:103911. 35248997 10.1016/j.ebiom.2022.103911 PMC8897583

[97] Raza MM Venkatesh KP Kvedar JC. Generative AI and large language models in health care: pathways to implementation. NPJ Digit Med 2024;7:62. 38454007 10.1038/s41746-023-00988-4 PMC10920625

[98] Ayers JW Poliak A Dredze M. Comparing physician and artificial intelligence chatbot responses to patient questions posted to a public social media forum. JAMA Intern Med 2023;183:589. 37115527 10.1001/jamainternmed.2023.1838 PMC10148230

[99] Mayol J. Transforming abdominal wall surgery with generative artificial intelligence. J Abdom Wall Surg 2023;2;12419. 38312403 10.3389/jaws.2023.12419 PMC10831645

[100] Ray TR Kellogg RT Fargen KM Hui F Vargas J. The perils and promises of generative artificial intelligence in neurointerventional surgery. J Neurointerv Surg 2024;16:4–7. 10.1136/jnis-2023-020353 37438101

[101] Rodler S Ganjavi C De Backer P. Generative artificial intelligence in surgery. Surgery 2024;175:1496–502. 38582732 10.1016/j.surg.2024.02.019

[102] Ray PP. Generative Artificial Intelligence (AI) and medical ethics: a symbiotic dance for the future. J Oral Maxillofacial Surg 2023;81:1457–59. 10.1016/j.joms.2023.09.015 38044013

